# The Smoky Impact of Nicotinic Acetylcholine Receptors on Testicular Function

**DOI:** 10.3390/jcm13175097

**Published:** 2024-08-28

**Authors:** Federica Barbagallo, Maria Rita Assenza, Filippo Torrisi, Alessandra Buonacquisto, Francesco Pallotti

**Affiliations:** 1 Department of Medicine and Surgery, Kore University of Enna, 94100 Enna, Italy; federica.barbagallo@unikore.it (F.B.); mariarita.assenza@unikore.it (M.R.A.); filippo.torrisi@unikore.it (F.T.); alessandra.buonacquisto@unikore.it (A.B.); 2 Endocrinology and Diabetology Unit, Hospital Umberto I, 94100 Enna, Italy

**Keywords:** cigarette smoking, Leydig cells, male fertility, nicotinic receptors, nicotine, Sertoli cells, spermatogenesis

## Abstract

Smoking habits (from classic cigarettes to e-cigarettes and heated tobacco) are a relatively common finding in the medical histories of couples referred to fertility centers. Tobacco smoke and e-cigarettes may deliver many substances with known harmful effects on both general and reproductive health, including nicotine. Nicotinic Acetylcholine receptors (nAChRs) form a heterogeneous family of ion channels that are differently expressed in different tissues. According to the homomeric or heteromeric combination of at least five different subunits (named from α to ε), they have peculiar pharmacological and biophysical properties. nAChRs respond to the neurotransmitter acetylcholine, which influences a number of physiological functions not restricted to neurons and plays an important role in the structure and function of non-neuronal tissues such as the testis. nAChRs are also the target of Nicotine, the active element responsible for tobacco addiction. This review summarizes recent findings on the involvement of nAChRs in testicular physiology, highlighting the effects of nicotine exposure observed in animal studies and clinical settings. We will discuss the latest data on fertility outcomes and the implications for understanding nAChR functions in reproductive health.

## 1. Introduction

Tobacco smoking is a relatively common habit with known harmful effects on general and reproductive health. WHO reports that roughly one-third of the global population >15 years (32.7%) were tobacco users in 2000, and, thanks to better knowledge about the risks associated with tobacco use and consistent efforts towards tobacco control in many countries, these numbers declined to approximately 22% and are projected to reduce further in the coming years [1].

Despite this reassuring data, cigarette smoking is reported in up to 37% of couples referring to fertility centers [2]. Most knowledge on the impact of smoking on male reproductive health derives from studies of patients smoking “conventional” tobacco cigarettes. In fact, tobacco combustion is known to produce thousands of gaseous (for example, carbon monoxide) and particulate compounds. These can be inhaled by smokers, and most of these have been recognized as potentially hazardous or frankly mutagenic (including heavy metals and nicotine, as well as carbon monoxide, aromatic hydrocarbons, etc.) [3]. Nicotine is an organic compound found in high concentrations in tobacco plants and other Solanaceae. It is an alkaloid with parasympathomimetic activity that can act through specific nicotinic acetylcholine receptors (nAChRs) [4]. It is considered among the most common and harmful substances contained in tobacco smoke since it can be absorbed quickly through the mucosae from the oral and respiratory tract. It has been elucidated that nicotine and its metabolites can reach the testes and exert effects on spermatogenesis and male fertility through various mechanisms, including oxidative stress, hormonal imbalance, inflammation, and apoptosis. Furthermore, the spreading of “new” smoking habits that entail the use of e-cigarettes and heat-not-burn (HNB) devices could not entirely remove the risk of smoking on male fertility since some substances, including nicotine, are still present [5,6,7]. Thus, this review aims to provide a state-of-the-art overview of nicotine’s effects on the testes with a specific focus on its putative mechanisms of action on testicular cells, exploring the role of nAChRs in testicular function and drawing on insights from both pre-clinical models and clinical evidence to understand their impact on male fertility. Understanding the mechanisms by which nAChRs influence testicular function could provide new opportunities for therapeutic intervention in male reproductive disorders.

## 2. Testicular Function and Nicotine

**Figure 1:**
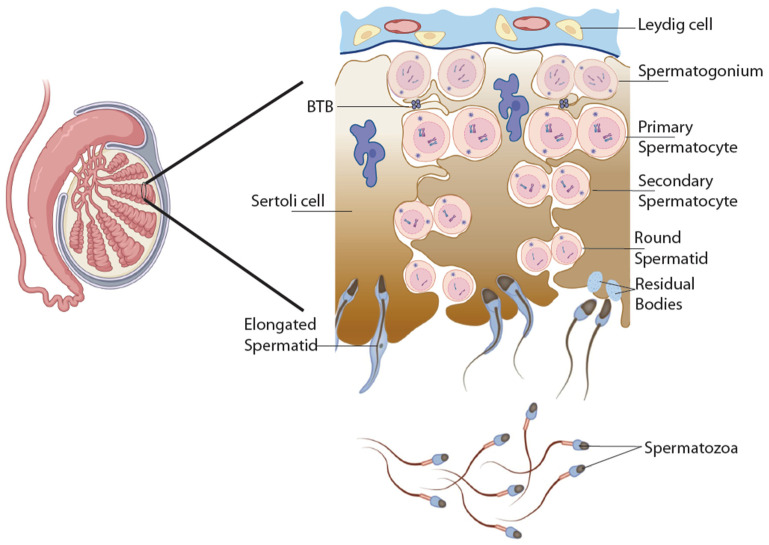
Schematic representation of spermatogenesis and spermiogenesis. Spermatogenesis takes place within the seminiferous tubules of the testes. Near the basal lamina (blue curved line), diploid primordial germ cells (spermatogonia) undergo mitosis, producing diploid primary spermatocytes. Primary spermatocytes move towards the lumen of the seminiferous tubules and begin meiosis I, resulting in haploid secondary spermatocytes. These secondary spermatocytes undergo meiosis II to produce haploid spermatids. Mature sperm cells (spermatozoa) develop from spermatids through spermiogenesis. Leydig cells, which produce testosterone, and a microvessel are located in the interstitial space. Sertoli cells form the blood–testis barrier (BTB), the tightest blood–tissue barrier in the mammalian body. Leydig and Sertoli cells create the cellular and hormonal environment necessary for spermatozoa development (created with BioRender.com).

Mammalian spermatogenesis is the intricate process through which sperm cells, or spermatozoa, are produced in the testes. This vital function is crucial for male fertility, enabling the production of millions of sperm daily to ensure successful reproduction. It comprises a complex differentiation process involving the interplay of different cell types and a series of cellular and biochemical metamorphoses occurring in seminiferous tubules (Figure 1) [8,9,10]. Somatic cells, i.e., Leydig cells located in the interstitium and Sertoli cells located within the tubules, create the cellular and hormonal environment necessary for spermatozoa development. The process begins with spermatogonia, diploid undifferentiated germ cells, located at the basement of the seminiferous epithelium. These cells undergo mitosis to produce type A spermatogonia, which serve as stem cells, and type B spermatogonia, which differentiate into primary spermatocytes. This phase ensures a continuous supply of germ cells for spermatogenesis. Primary spermatocytes enter the first meiotic division (Meiosis I) to form secondary spermatocytes, each containing half the number of chromosomes. These secondary spermatocytes then undergo the second meiotic division (Meiosis II) to produce haploid spermatids. Meiosis ensures genetic diversity and reduces the chromosome number by half, preparing the cells for fertilization. Spermatids are transformed into spermatozoa by the differentiation process named spermiogenesis. This phase involves significant morphological changes, including the development of the acrosome, which contains enzymes necessary for penetrating the egg, condensation of the nucleus, and formation of the flagellum, which provides motility. The cytoplasm is reduced, and the mitochondria are arranged in the tail mid-piece to supply energy for movement. Spermatozoa are then transported to the epididymis, where they undergo further maturation, gaining motility and the ability to fertilize an egg. Mature sperm are then stored in the epididymis until ejaculation, when they travel through the vas deferens, mix with seminal fluid from the seminal vesicles, prostate gland, and bulbourethral glands, and are expelled through the urethra.

### 2.1. Structure and Function of nAChRs

nAChRs are a subtype of acetylcholine receptors, a class of ligand-gated ion channels that play crucial roles in the nervous system by mediating fast synaptic transmission. nAChRs are pentameric complexes composed of different combinations of at least five different subunits (named from α to ε) arranged symmetrically around a central pore [11]. For example, the nAChRs expressed in the muscle are composed of either (α1)2 β1 γ δ subunits in fetal tissue or (α1)2 β1 γ ε subunits in adult tissues. These subunits belong to a larger family encoded by at least 17 different genes, giving rise to various receptor subtypes with distinct functional properties such as ion selectivity and kinetic behavior [11,12,13]. Neuronal-type nAChRs are composed solely of α and β subunits, but subunit association is promiscuous, generating a high level of functional diversity in terms of pharmacological specificities, channel permeability, and kinetics. Activation of nAChRs occurs upon the binding of the neurotransmitter acetylcholine (ACh) or exogenous agonists such as nicotine. This binding induces a conformational change that opens the ion channel, allowing the flow of cations, particularly sodium (Na^+^) and calcium (Ca^2+^), across the cell membrane. This ion flux leads to depolarization of the membrane and subsequent activation of intracellular signaling pathways. In addition to their well-characterized role in the nervous system, nAChRs are widely distributed in non-neuronal tissues, including the immune system, epithelial cells, and reproductive organs [14,15,16]. Their presence in these tissues suggests that nAChRs may have broader physiological roles, potentially influencing cellular processes such as proliferation, differentiation, and apoptosis.

### 2.2. Expression of nAChRs in the Testes

The components of the so-called non-neuronal nicotine system have been identified in the male reproductive tract. ACh and its transporters (choline transporter 1 -ChT1- and the vesicular acetylcholine transporter -VAChT), the choline acetyltransferase (CHAT), and the nAChRs were first found in the parenchyma of rat testis [17,18]. The expression of nAChRs in testicular tissue has been documented in several studies, allowing us to hypothesize that these receptors may play a significant role in testicular function. The detection and quantification of these receptors typically involve techniques such as immunohistochemistry, reverse transcription-polymerase chain reaction (RT-PCR), and Western blotting. Studies have shown that nAChRs are expressed in all the cell types within the testes, including Leydig, Sertoli, and germ cells.

The analysis of non-neuronal cells in the parenchyma of rat testes revealed that the highest levels of expression account for mRNA of α4-, α7-, α5- and α9- and β(1-3)- subunits [18]. A further characterization highlighted that spermatogonia and spermatocytes were the subpopulations expressing the α4-subunit. The mRNA expression of this subunit has been confirmed by several other studies [19,20], but its protein expression has not been verified in the spermatogonial pool [21], making it difficult to study its role in this compartment. The α7 subunit is a common component of nAChRs and is known for its role in calcium signaling. Indeed, ACh receptors consisting solely of cholinergic receptor nicotinic alpha 7 (CHRNA7) subunits (i.e., homomeric) are highly permeable to calcium ions compared to the heteromeric form [18,22]. The expression of the α7-subunit of nAChRs was detected in spermatogonia, spermatocytes, differentiating germ cells, and somatic Sertoli cells within the seminiferous tubules [18,22]. Nicotine antagonists (hexamethomium) appear to partially reverse the inhibition of testosterone release by Percoll-purified Leydig cells of the rat, suggesting that these cells are able to express nAChRs [23]. Ge et al. demonstrated that rat-differentiated Leydig cells possess high levels of acetylcholine receptor nicotinic α4 (CHRNA4) [24]. Recently, the ε subunit (AChRε) was found to have a higher level of mRNA expression in mouse testes than the other subunits [25], with a restricted expression in elongated spermatids from step 12 onwards. Further evidence comes from the localization and function of nAChR in spermatozoa. Immunofluorescence and immunogold staining analysis for the α7-subunit on sperm isolated from different species visualized the protein localization on the tail and post-acrosomal area of human spermatozoa [26]. Using a fluorescent alpha-bungarotoxin-tetramethyl-rhodamine conjugate that binds a range of nicotinic acetylcholine receptor subunits and comparing WT and null CHRNA7 gene mice revealed that the α7-subunit is present on the midpiece of mouse sperm [27]. In the same year, Kumar and Meizel demonstrated that human sperm also express α3-, α5-, α9-, and β4- nAChR subunits and that they localize to the midpiece, neck, and post-acrosomal regions and that the α9 subunit in particular was restricted in the mid-piece region of sperm [28]. The expression of these isoforms was questioned later on, leaving the α7-subunit the only one that was certainly expressed [29]. Immunocytochemistry analysis recently revealed that AChRε is also expressed in spermatozoa [25]. A possible role of nAChR can be deduced from sperm stimulation with micromolar concentrations of ACh; this stimulation appears to initiate the acrosome reaction (AR) in both capacitated human and mouse spermatozoa [30], and this effect is particularly mediated by the α7 nAChR [30,31,32] by triggering the Ca^2+^ influx required for AR [30,31,33]. Further evidence of nAChR involvement in fertilization has been acquired from α7 null mice. While these mice are potentially fertile, a degree of reduction in fertility caused by impaired sperm kinetics and impaired hyperactivated motility has been described [27,34].

## 3. Preclinical Models for Studying Nicotine’s Effects on Testicular Function

Various preclinical models, including rodents (rats and mice) and other small mammals, have been employed to study the impact of nicotine on spermatogenesis. These models provide valuable insights due to their physiological and genetic similarities to humans, ease of handling, and usefulness in isolating nicotine’s contribution among all the substances present in cigarettes [35] since, as mentioned, they contain, besides nicotine, several toxic chemicals such as benzopyrene, dimethylbenzanthracene, naphthalene, methylnaphthalene, polycyclic aromatic hydrocarbons (PAHs), and cadmium [36].

### 3.1. Animal Models of Nicotine Exposure

Nicotine is oxidized within the body to its metabolite cotinine, which presents an extended half-life and can affect the male reproductive system and fertility [37,38]. Nicotine exposure has been shown to induce significant histological changes in testicular tissue, including decreased testicular weight, reduced seminiferous tubule diameter, and disruption of the normal architecture of the seminiferous epithelium. Rats given nicotine presented a thickening of the tunica propria, likely due to an increase in the collagen fibers [37,39]. Fetal and neonatal nicotine exposure induces morphological differences in the testis, characterized by a higher number of degenerated tubules, in rats aged 7 weeks compared to the control [40]. This was highlighted by the finding that LDH and alkaline phosphatase activity was increased in nicotine-exposed rats, underlining the ongoing degenerative process. In rats exposed to nicotine 60 and 90 days postpartum (dpp), nuclear pyknosis, irregular chromatin condensation, and intense cytoplasmic eosinophilia were observed, suggesting that cell death was caused by necrosis [41]. Nicotine could also cause other morphological changes, such as the detachment of the seminiferous epithelium from the basal lamina and its folding into the lumen, likely through the reduction of vimentin expression and subsequent weakening of the cellular junctions of the exposed rats. To the same extent, cigarettes-exposed rodents, with increased severity dependent on time and dose, exhibited smaller seminiferous tubules accompanied by atrophy, degeneration of tubules, decrease of the spermatogenic cell layer, and an enlargement of interstitial areas with a conspicuous reduction in Leydig cells [42,43]. The latter has also been observed in nicotine-treated rats [39]. In particular, qPCR and Western blotting following nicotine administration were associated with reduced mRNA levels and protein expression of key steroidogenesis enzymes (CYP11A1, 3β-HSD1, CYP17A1, 17β-HSD3, NR5A1). Sertoli cells were also affected, exhibiting intracellular cavities, with malformed nuclei characterized by condensed chromatin. Interestingly, nicotine damages the blood–testis-barrier (BTB), inducing degeneration of the junction between the cells, likely by inducing ferroptosis [44]. Additionally, nicotine can decrease SOX9 expression, a Sertoli cell marker that exhibits the circadian rhythm in the testis and can also disturb its oscillation. Given that Sertoli cells are important actors of the BTB, it has been suggested that nicotine may destroy the BTB’s integrity by disturbing the circadian clock system of Sertoli cells [44]. Nicotine adversely affects the population of germ cells at various stages of development. Preclinical studies have demonstrated a reduction in the number of spermatogonia, spermatocytes, and spermatids following nicotine exposure. This decline in germ cell numbers suggests that nicotine interferes with the proliferation and differentiation processes essential for normal spermatogenesis. The mechanism of damage by nicotine could be explained in different ways that are not always easy to dissect: (1) an indirect action on the hypothalamic-pituitary-testicular axis that affects hormonal production and/or (2) a direct action on testicular cells. It has been shown that nicotine exposure caused seminiferous tubular derangement, sloughing off germ cells from the germinal epithelium with the disintegration of spermatocytes and spermatids, resulting in the disruption of spermatogenesis [45]. Nicotine delayed the maturation of gonocytes into spermatogonia [22]. The mechanism of damage may involve nicotine’s direct effect on Sertoli cells since the latter promotes the maturation of gonocytes to spermatogonia by providing factors like retinoic acid, platelet-derived growth factor-BB and its receptor, and Anti-Mullerian Hormone (AMH). In vitro studies with Sertoli cells isolated from prepubertal animals exposed to nicotine have shown functional alterations, including reduced mRNA expression and protein levels of AMH and Inhibin-B, impaired FSH-r, and downregulation of Bcl2 [46]. Additionally, nicotine may act directly on gonocytes. The higher optical density (OD) of α7-nAChR observed in animals exposed to nicotine at 3, 7, and 10 dpp, where gonocytes population is predominant, suggests a direct effect on germ cells. Activated nAChR increases ion influx, primarily Ca^2+^ [22], and there is in vitro evidence suggesting that increased intracellular Ca^2+^ induces mitochondrial membrane depolarization, leading to the release of proapoptotic factors by activating the permeability transition pore and compromising mitochondrial membrane integrity.

Aydos et al. [37] used ultrastructural analysis of the testis of rats exposed to nicotine in adulthood and reported anomalies in spermatids. Moreover, an abundant cytoplasmic area was found in the round spermatids of adult rats exposed to nicotine during their embryonic/fetal life [41]. It has been proposed that these effects may be associated with the failure to remove residual cytoplasm in sperm, which can lead to defects in sperm function. Finally, binucleated formations in round spermatids were observed in treated animals, which is probably due to defects in spermatids’ cytoskeleton and/or cytokinesis. Dai et al. performed two-dimensional gel electrophoresis and mass spectrometry analysis to identify differentially expressed proteins from the testes of mice exposed to nicotine daily compared to controls, and the analysis of the data allowed the identification of proteins mainly involved in actin cytoskeleton regulation. In particular, PFN1, a central regulatory factor of the cytoskeleton, was up-regulated in nicotine-treated mice, specifically in elongated spermatids [47]. Later on, it was proposed that nicotine induces apoptosis in germ cells and that it was favored by the decline of telomerase activity caused by the overexpression of NM23-H2/nucleoside diphosphate kinase B (Nme2) [48].

### 3.2. Mechanisms of Spermatogenesis Impairment from Nicotine Exposure

Sperm abnormalities have also been associated with nicotine administration. In general, nicotine is associated with reduced fertilization capabilities of sperm, possibly caused by reduced sperm count and concentration, impaired sperm kinetics, and increased abnormalities (head and tail in particular) [49,50]. These sperm alterations in treated animals are likely caused by a variety of mechanisms.

#### 3.2.1. Oxidative Stress and Apoptosis

One of the proposed mechanisms by which nicotine impairs spermatogenesis is through the induction of oxidative stress. Nicotine metabolism generates reactive oxygen species (ROS) and lipid peroxidation [51,52], which can cause oxidative damage to cellular components, including DNA, proteins, and lipids. Increased levels of oxidative stress markers, such as malondialdehyde (MDA), and decreased activities of reduced glutathione (GSH) metabolism-regulating enzymes, such as glutathione reductase activity, glutamyl transpeptidase (c-GT), glutathione peroxidase (GPx), glutathione reductase (GR), and glucose-6-phosphate dehydrogenase (G-6-PDH), have been observed in the testes of nicotine-exposed animals [52,53,54,55], triggering apoptosis in germ cells [56]. Studies have reported elevated expression of pro-apoptotic proteins (e.g., p53, Bax, cleaved Caspase 3) and time-dependent decreased expression of anti-apoptotic proteins (e.g., BCL2 Like 2-BCL-2L2) in the testes of nicotine- or cigarette-smoking-treated animals [43], which adversely affects the germinal epithelium in adult rats. This imbalance promotes germ cell apoptosis through the mitochondrial apoptotic pathway, further compromising spermatogenesis.

#### 3.2.2. Hormonal Imbalance

Preclinical models have shown that nicotine exposure disrupts the hormonal balance necessary for normal spermatogenesis, even if a consensus on the increase or decrease of the different hormone levels is still missing. For instance, nicotine has been shown, by some groups, to significantly decrease serum levels of FSH and significantly increase the circulating levels of prolactin and LH [50]; a lower level of LH and a higher level of prolactin were observed in the nicotine-treated animal group compared to controls in another study [54]. Others verified that the indirect absorption of nicotine during lactation causes changes in the hypothalamic–pituitary–thyroid axis, leading to a significant increase of both FSH and LH plasma levels in adult rats exposed to nicotine [41]. Most of the research findings agreed that nicotine decreases the level of testosterone [38,39,57]. The cause of this reduction was attributed to different causes. Firstly, it has been proven that nicotine is able to induce autophagy in Leydig cells; indeed, the expression of autophagy-related genes, such as Beclin and LC3, was increased after nicotine exposure. The autophagy in Leydig cells was set by the hyper-methylation of the T-cell leukemia/lymphoma protein1 (TCL1) promoter region that, in turn, reduces TCL1 protein expression and impedes the activation of the anti-TCL1-mTOR-autophagy signaling pathway [58]. Another group related nicotine’s effect to its ability to inhibit Leydig cell differentiation and maturation by influencing the Hedgehog signal pathway [59]. Finally, a direct effect on in vitro-cultured Leydig cells highlighted a negative effect of this compound on steroidogenic capacities by inhibiting the expressions and activities of a steroidogenic enzyme such as the testicular steroidogenic acute regulatory (StAR) protein, side cleavage enzyme, 3β-hydroxysteroid dehydrogenase 1, 17 alpha-hydroxylase, 17, 20-lyase, and steroidogenic factor 1 [60,61,62].

#### 3.2.3. Inflammatory Response

Nicotine can also elicit an inflammatory response in the testes. Studies have reported increased expression of pro-inflammatory cytokines (e.g., tumor necrosis factor-α (TNF-α), interleukin 1-beta (IL-1β)) and the activation of inflammatory pathways (e.g., nuclear factor-κB, (NF-κB)) in the testes of nicotine-exposed animals [63,64]. Moreover, spermatogonia and primary spermatocytes stained positive for Cyclooxygenase-2 (COX-2) when animals were exposed to nicotine [64]. Similar findings have been reported by Kushwaha and Jena when diabetic rats were exposed to nicotine. Increased levels of NF-κB, COX-2, and TNF-α expression were reported in testes isolated from these animals [65]. Inflammation can further damage testicular tissue and impair the spermatogenic process.

## 4. Clinical Evidence of Nicotine’s Effects on Testicular Function

The scientific literature on data regarding the effects of smoking habits on male reproduction and semen quality is abundant [2,66,67]. Despite the deleterious effects of cigarette smoke and nicotine being widely accepted, most studies focus mainly on conventional cigarette use; therefore, the impact of alternative tobacco products and e-cigarettes on reproductive health is definitely understudied [68].

### 4.1. “Conventional” Cigarettes

Most of the harmful impact of cigarette smoking on male fertility is known to be associated with heavy metals—Cadmium (Cd) and Lead (Pb) in particular—which are well-studied reproductive toxicants in cigarette smoke. Tobacco leaves accumulate a considerable amount of Cd, which smokers inhale and accumulate mainly in the liver and kidneys but also in the testes [69]. Cd can be detected in the seminal plasma of heavy smokers, and there is evidence of how this metal can impair both testicular and hypothalamus–pituitary–gonadal axis function through inflammation and oxidative stress, endothelial damage, BTB disruption, and apoptosis [69,70]. A recent study reported the association between Cd content in semen and male infertility [71]. Inhaled Pb particles can interfere with calcium-dependent cellular processes and induce further cellular damage through the formation of ROS. Pb-induced damage may affect the brain, kidneys, cardiovascular and gastrointestinal systems, and the reproductive system by impairing reproductive hormone levels and semen quality [72]. Although several variables (for example, possible pollutant co-exposure, absorption, and tissue distribution) could affect the results of available observational studies, detrimental effects of heavy metals in cigarette smoke have been robustly associated with reproductive damage, and Pb and Cd levels in semen and urine are associated with oxidative stress, semen quality impairment, and infertility [73,74]. Aside from heavy metals, cigarettes and their smoke contain a significant amount of nicotine. It is among the most studied compounds, and its presence in the seminal plasma of smokers has been previously described [75]. Nicotine derived from burnt tobacco is carried proximally on tar droplets (also called particulate matter), which are inhaled. Despite the well-known travel of nicotine and its metabolites from lung absorption to the male genital trait, there are contrasting data on their in vivo effects. An important reason is that, unlike controlled experiments and animal models, in vivo studies cannot completely clarify whether the described effects are nicotine-related or caused/mediated by other metals or chemicals (or even mixtures) contained in cigarette smoke. Furthermore, another layer of difficulty in the evaluation of the effects of nicotine on human testicular function is the variability of smoking habits among individuals, including the utilization of “new” devices.

### 4.2. New Smoking Habits

While e-cigarettes and HNB tobacco devices are advertised as reduced-risk products due to the reduced number of substances present in their smoke (and thus potentially inhaled), some harmful substances are still present in different concentrations [3].

In general, e-cigarettes produce an aerosol containing propylene glycol, glycerin, water, and ethanol; other substances, nicotine in particular, but also different substances that add specific flavors to the mixture, are added to increase the pleasantness for the user [76]. HNB tobacco products, instead, are designed to heat tobacco at around 350 °C, and inhalation of the “smoke” produced allows for the efficient delivery of nicotine; the tobacco sticks used in these devices are generally enriched with flavors, as with e-cigarette liquids [6].

It is particularly important to stress the fact that most products may still contain nicotine, which can be absorbed and can be found in the bloodstream in concentrations comparable to or even higher than that found in classic cigarettes [77,78]. Nicotine absorption and the detection of its metabolites in 24-h urine collection in different groups of tobacco and nicotine products users highlight that classical cigarette smoking is associated with a higher daily nicotine absorbed dose compared to e-cigarettes and HNB devices; however, significant inter-individual variations are possible depending on different smoking habits [79]. Also, the concentration of nicotine contained in the device may impact absorption. For example, a recent paper highlighted that e-cigarettes using nicotine salts are associated with high nicotine concentrations in the bloodstream: in a median of 2.0–2.5 min, blood nicotine reaches the Cmax, and a 40 mg/mL nicotine salt formulation can potentially deliver an amount of nicotine comparable to that in conventional cigarettes [80]. Daily dose and quickness of nicotine absorption may be other factors involved in the development of addiction (and maintenance of the smoking habit) since nicotine has both direct and indirect effects on the central nervous system through other neurotransmitters [4]. These, in turn, may have a role in addiction to tobacco and e-cigarettes, thus reinforcing the effects on reproductive health and testicular function.

### 4.3. Clinical Data on Smoking Habits and Male Reproductive Health

Most of the available clinical studies on the effects of smoking habits on male reproductive parameters are observational in nature. It should also be noted that, frequently, consumers simultaneously utilize conventional cigarettes and e-cigarettes or other tobacco products (so-called “dual users”), implying further difficulty in evaluating the effects of a single product. Another difficulty in evaluating data on this topic is that most papers generically categorize subjects only into current, former, and non-smokers. While this allows a certain degree of data reproducibility, it does not account for different types of cigarettes/devices, tobacco “doses”, or a consistent measure of how much a subject inhales and allows the introduction of recall biases from the subjects. Moreover, a critical limitation of in vivo observational studies on smoking habits’ effects on male reproduction is that it is challenging to elucidate the effects of single toxicants on specific fertility factors (semen parameters, chromatin integrity, fertilization, etc.). Nonetheless, studies have focused on peculiar aspects of male reproduction.

#### 4.3.1. Semen Quality

Experimental exposure of semen samples to cigarette smoke causes a significant reduction of sperm motility [81]. These negative effects on spermatogenesis have been mostly attributed to oxidative stress caused by the increase of ROS induced by tobacco combustion and smoke breathing. In vitro studies have shown that nicotine and its metabolites may be involved in a dose- and time-dependent way—arbitrary nicotine concentrations have been associated with sperm kinetics impairment, reduced mitochondrial membrane potential, and sperm chromatin damage [29,81,82]. In vivo human observational studies repeatedly observed that substances contained in the smoke from cigarettes can negatively affect semen volume, sperm parameters, and mitochondrial activity, therefore harming reproductive health and, consequently, fertilization both in vivo and in vitro [2,83,84]. In 2016, Sharma et al. performed a comprehensive metanalysis of available studies (reporting 20 studies and a total of 5865 participants) clearly indicating that cigarette smoking was significantly and negatively associated with sperm count, motility, and normal morphology; moreover, the deleterious effect of exposure to cigarettes was more pronounced in moderate/heavy smokers and in infertile participants [2]. This underlines a dose-dependent effect but also suggests that preexisting andrological diseases (associated with infertility) could render subjects more vulnerable to the deleterious effects of smoke. Regarding e-cigarettes, the evidence is scant. A recent cross-sectional study investigating the smoking habits of young Danish adults highlighted that electronic and conventional cigarettes had a similar reduction of total sperm count compared to non-smokers, even when accounting for potential confounders [85]. Nicotine could also affect semen quality by affecting other components of the male genital trait. Lotti et al. highlighted how, among men from infertile couples, current smokers had lower semen ejaculate and seminal vesicle volume both before and after ejaculation compared to non-smokers [86]. This is an interesting aspect of smoke reproductive toxicology: seminal vesicles contribute to cholinergic signaling in seminal plasma [87], and nAChR activation is known to modulate smooth muscle cell activity in the genital trait [88,89]. Thus, it is possible that chronic exposure to nicotine could correspond to an abnormal cholinergic tone in seminal vesicles, which could result in impaired contraction. Since seminal vesicles contribute roughly two-thirds of the final semen volume, this anomalous vesicle contraction could justify the reduction observed in infertile smokers. However, it must be noted that these effects could also be mediated by other chemicals in cigarette smoke.

#### 4.3.2. Hormone Profile

A meta-analysis including 22 observational studies (for a total of 13,317 adult men aged 18–61 years) indicated an increase in testosterone levels in smokers compared to non-smokers (~1.5 nmol/L) and similar results in smoking women [90]. A similar increase in total and free testosterone has been confirmed in a recent observational study in daily cigarette smokers when compared to non-smokers, but this was not observed in e-cigarette users [85]. This is interesting as it is in apparent contrast with animal studies’ findings. It can be speculated that in experimental models, animals are administered nicotine doses that, related to body size, are much higher than those men inhale through smoke, exposing the animal to a greater degree of damage to reproductive tissues. In humans, it is known that a possible biological explanation lies in the androgen disposal pathway, which is in common with nicotine and its metabolites—testosterone, cotinine, and trans-3′-hydroxycotinine are inactivated and eliminated through glucuronidation by the liver UDP-glucuronosyltransferase enzyme. Nicotine may competitively inhibit this pathway, justifying the mild circulating androgen increase [90]. This mechanism could apply to both conventional cigarettes and other products due to their capability to deliver significant amounts of nicotine, but so far, robust observational evidence for the latter is lacking. Nonetheless, the direct effects of alternative smoking products on the HPG axis should not be excluded a priori. For example, Shah et al. proved that, while circulating testosterone was increased in both smokers and smokeless tobacco users compared to a control group, kisspeptin levels were increased only in subjects using alternative tobacco products [91]. Even smokeless and tobacco-free products may allow the absorption of significant amounts of nicotine, as demonstrated by the measurement of urinary cotinine levels [92]. Since there is insufficient evidence on e-cigarettes and HNB devices, this should be considered an area to focus on, as products associated with new smoking habits may allow for the better absorption of substances that may cause increased pharmacological activity of nicotine and other substances both in the central nervous system and in the gonads.

#### 4.3.3. Sperm Chromatin Integrity and Oxidative Stress

**Figure 2:**
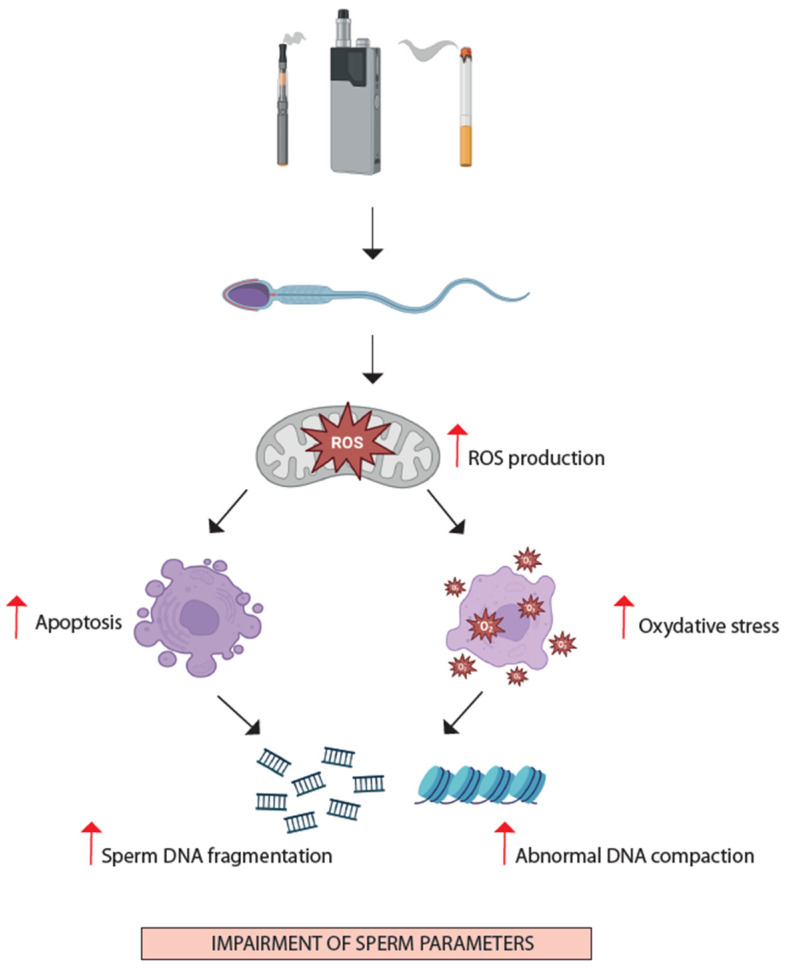
The figure summarizes the known effects of tobacco products on semen quality. Tobacco smoke is known to increase the formation of reactive oxygen species (ROS), leading to oxidative stress. The excessive buildup of ROS causes lipid peroxidation and DNA strand breaks, resulting in altered sperm parameters and functions. This increase in ROS levels can also trigger apoptosis by raising the levels of pro-apoptotic cytokines, which further contributes to sperm DNA damage and impaired male fertility. Furthermore, tobacco smoke has been shown to decrease nuclear protamine levels and protamination (created with BioRender.com).

Chronic exposure to cigarette smoking has also been associated with impaired germ cell differentiation, especially regarding chromatin packaging and integrity. As previously discussed, this is generally associated with increased sperm oxidative stress (Figure 2) [67,93]. Oxidative stress is a key factor contributing to male infertility, particularly in smokers, where the balance between ROS production and antioxidant defenses is disrupted. There is evidence that both conventional cigarettes and alternative products are associated with increased biomarkers of DNA oxidation, such as 8-hydroxy-2’-deoxyguanosine (8-OHdG), compared to non-smokers [94,95,96]. This was also investigated in the seminal fluid, where increased 8-OHdG levels can be detected in the seminal plasma of cigarette smokers [97]. Sperm DNA integrity is essential for correct sperm functioning and proper transmission of genetic material to the embryo—increased sperm DNA fragmentation (SDF) is linked to impaired spermatogenesis, aberrant apoptosis, poor embryo quality, and recurrent pregnancy loss [98]. Smoking is considered among the major causes of sperm chromatin damage along with obesity and other modifiable lifestyle factors, male accessory gland infections, varicocele, and neoplastic diseases [99]. Most studies indicate an increase in SDF in smokers [100], with slight differences depending on the method used to perform the measurement. Tobacco constituents (including heavy metals and nicotine) could lead to an unbalanced oxidant environment with increased ROS leading, in turn, to DNA damage and germ cell apoptosis [101]. Interestingly, a recent study showed that users of oral smokeless tobacco do not have significant differences in sperm DNA fragmentation compared to non-users [92]. This could indicate either that tobacco constituents other than nicotine are responsible for the oxidative stress and DNA-damaging effects of smoke or that this effect could be more pronounced in infertile subjects than in the general population. In either case, more research is warranted to elucidate this aspect. Hammadeh et al. have shown that smoking could influence the protamination process [102]. Protamines (PRM) are key proteins in spermatozoa nuclei, allowing sperm DNA compaction and protection. Smokers appear to have reduced Protamine-2 (PRM2) expression, resulting in an abnormally increased PRM1/PRM2 ratio; in turn, this is associated with abnormal chromatin compaction and increased sperm DNA fragmentation levels [102]. This indicates how oxidative stress related to cigarette smoking impacts negatively on the sperm protamination process and DNA integrity, thus potentially impairing male fertility at the sperm nuclear quality level. Cigarette smoking has also been described as a hindrance to infertility treatments. Colacurci et al. have detected that idiopathic infertile men treated with 3 months of subcutaneous FSH injection to stimulate spermatogenesis had a poorer response in terms of sperm DNA integrity reduction if they were smokers compared to non-smokers [103].

#### 4.3.4. Epigenetic Modifications

It is known that the environment, including lifestyle habits such as smoking, could affect the epigenetic response of the individual, and the most known epigenetic modification is DNA methylation, which effectively “silences” gene transcription. In general, methylation of sperm DNA, either as global methylation or methylation of specific imprinted genes, has been associated with male infertility, oligozoospermia, and sperm quality impairment [104]. Smokers of conventional cigarettes have been shown to have altered sperm global DNA methylation, which is, in turn, associated with sperm chromatin condensation defects and SDF [105]. Another relevant epigenetic response is the modifications of the expression of small non-coding RNAs such as miRNAs. Sperm miRNA expression is of great interest in reproductive medicine, and it has been highlighted that the smoking habit induces differential expression of several miRNAs in smokers compared to non-smokers; in particular, these miRNAs appear to be involved in pathways regulating cell survival and apoptosis, both critical aspects of spermatogenesis and embryo development [106]. There is a lack of data about the germline epigenetic effects of e-cigarettes and HNB devices, leaving a significant knowledge gap. Also, the direct influence of nicotine cannot be fully established as the smoke mixture also includes other toxicants. In any case, it should be noted that since this epigenetic modification occurs in the germinal line, it can impact transgenerationally on the health of the offspring [107].

## 5. Future Perspectives

Considering all the evidence collected, the nAChRs appear to play a major role in testicular function and represent an emerging area of research with significant implications for reproductive health that is as yet understudied. A full characterization of the specific molecular pathways through which specific nAChR subtypes regulate testicular functions (such as spermatogenesis, hormone production, and the maintenance of the blood–testis barrier) is needed and should be the focus of future studies. On the one hand, a better understanding of these mechanisms could lay the basis for the identification of reproductive disorders associated with nAChRs dysfunction; on the other hand, improved knowledge of nAChRs pathophysiology would allow us to develop new targeted therapies for male infertility. It has been recently outlined that nAChRs are important targets in a variety of pathological conditions, and several substances acting as receptor modulators have been investigated as potential new medications for chronic obstructive pulmonary disease and asthma and possibly for neurological conditions associated with nAChRs dysfunctions [108]. Although investigations of testicular functions are scant, this could prove to be an interesting field of research. While preclinical studies have provided insights into the detrimental effects of nicotine on sperm quality and testosterone levels, translating these findings to clinical practice is essential. This will involve not only refining our understanding of how nicotine interacts with nAChRs in human testicular tissue but also exploring potential therapeutic interventions to mitigate these effects. As research progresses from the bench to the bedside, it is anticipated that new diagnostic tools and treatment strategies will emerge, offering better outcomes for patients affected by nicotine-induced reproductive dysfunction. Furthermore, the impact of nicotine on testicular health warrants deeper investigation, even in light of the introduction and dissemination of “new” smoking habits involving devices such as electronic cigarettes and HNB devices. These have been demonstrated to deliver nicotine efficiently into the bloodstream, but most of the knowledge on the effects of smoke still relies on data from conventional cigarettes. Future clinical investigations should aim to investigate the presence of semen inflammatory and oxidative biomarkers (such as 8-OHdG) in users of new devices in order to provide valuable insights into the extent of oxidative damage and to inform potential interventions to mitigate the adverse effects of smoking on male reproductive health. Since most of the damage to reproductive function induced by nicotine and smoke is associated with increased oxidative stress, antioxidant supplements through diet or supplements could be considered a treatment of choice. Despite generally positive effects on semen parameters [109], there is a lack of consensus on these treatments for the management of male factor infertility, especially in the case of smokers, as literature data lack quality and the evidence is controversial. Finally, research should also be pointed toward the epigenetic response in terms of DNA methylation and miRNA expression due to the extreme significance of these aspects for reproduction. Furthermore, it is known that nicotine itself can modulate miRNA expression, mediating nAChRs signaling and tissue-specific nicotine effects [110]. Currently, there are no data on this nicotinic signaling modulation on testicular function, and filling this gap in knowledge could assist in developing treatment strategies to prevent the harmful testicular effects of nicotine.

## 6. Conclusions

**Figure 3:**
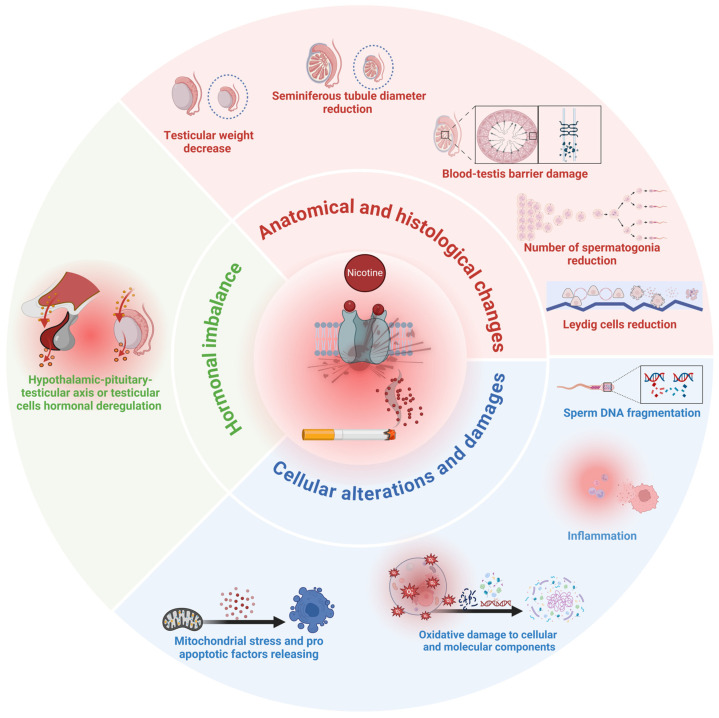
Summary representation of main known effects of nicotine on testicular function (created with BioRender.com, accessed on 21 August 2024).

There is broad consensus that conventional cigarettes can significantly affect reproduction at multiple levels. Upcoming evidence, although still conflicting, appears to indicate that the “new” smoking habits (e-cigarettes and HNB devices, in particular) also share several risks for reproductive health. Nicotine is a common constituent of the smoke produced by all these devices and cigarettes. Although it has a less direct effect than common toxicants of cigarette smoke (like Cd and Pb), its quick absorption, distribution over the bloodstream up to the testes and seminal plasma, and the presence of nAChR in germ and somatic cells at various stages of differentiation, represent the biological background of its action, as demonstrated in animal and in vitro models (Figure 3).

Despite the difficulties of conducting in vivo observational studies, a putative harmful effect of nicotine can be hypothesized. In particular, literature data clearly indicate that nicotine may play a causative role, either directly or indirectly, in altering:

- Hormone axis: alterations of blood testosterone and gonadotropin levels.
- Testicular histology: BTB damage, Leydig cell dysfunction, alteration of seminiferous tubules.
- Spermatozoa: increased mitochondrial stress and apoptosis, increased oxidative stress and inflammation, increased SDF.

Therefore, a clinical andrologist and reproductive health specialist should investigate the patient’s smoking habits through a careful collection of their medical history. The utilization of the so-called reduced-risk products should not lower the attention paid to these aspects, as many of these alternative products may be able to deliver amounts of nicotine comparable to conventional cigarettes and possibly have damaging effects on sperm parameters and DNA integrity.

## Data Availability

Not applicable.
